# Armored brain in a young girl with a syndromal hydrocephalus

**DOI:** 10.1007/s00701-016-2991-1

**Published:** 2016-10-25

**Authors:** Ilaria Viozzi, Kirsten van Baarsen, André Grotenhuis

**Affiliations:** Department of Neurosurgery, Radboud University Medical Centre, Geert Grooteplein Zuid 10, 6525 GA Nijmegen, Gelderland The Netherlands

**Keywords:** Armored brain, Matrioska effect, Bilateral chronic ossified hematoma, Shunt complication

## Abstract

The authors present a case of a young girl affected by a syndromal hydrocephalus who developed a bilateral ossified chronic subdural hematoma with the typical radiological appearance of "the armored brain". Bilateral calcified chronic subdural hematoma is a rare complication of ventriculoperitoneal shunt. There is controversy in the treatment, but most published literature discourages a surgical intervention to remove the calcifications.

## Introduction

**Fig. 1:**
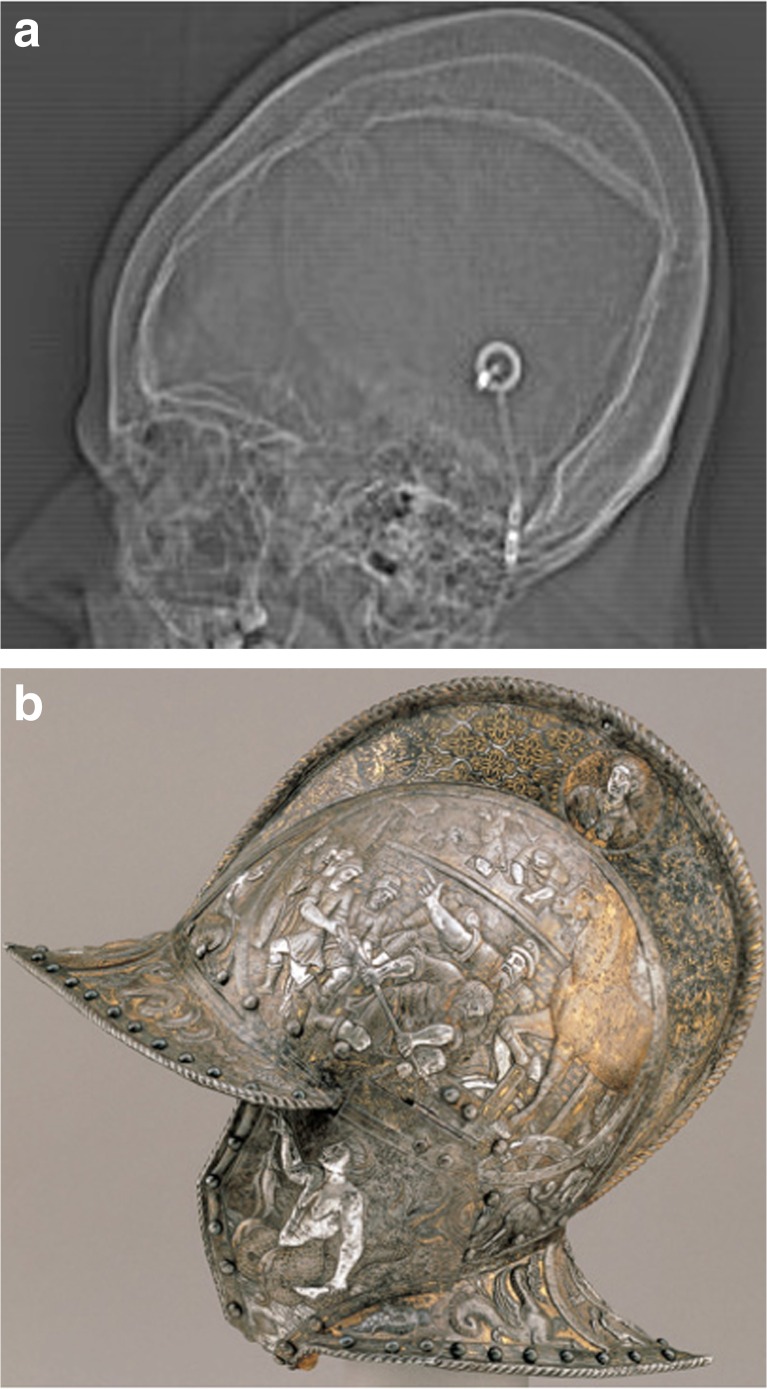
Toposcan CT: double calcification of the skull (**a**). Note the similarity with an armored helmet (**b**)

‘Armored brain’ is a term used to describe a rare clinical condition of bilateral ossified chronic subdural hematoma or hygroma [1]. The word armored derives from Latin *armatura* and later from Anglo-French *armour (e)* used to describe a metal sheathing or protective covering [2] (Fig. 1a). This condition is diagnosed by the presence of hyperdense outer and inner rims separated by a slightly hypodense area on CT [3]. This phenomenon is pathognomonic and it is also known as ‘the matrioska effect’ [4]. The treatment is controversial, but surgical therapy might be an option if the condition leads to mass effect and raised intracranial pressure [5].

The authors present a case of bilateral ossified hygroma in a 15-year-old child under treatment because of a syndromal postnatal hydrocephalus with a ventriculoperitoneal shunt.

### Case report

The patient was born with a chromosomal 6q deletion, a rare genetic syndrome with a broad spectrum of symptoms and presentations. Since birth, she has been affected with hydrocephalus, which was treated at 1 month of age by ventriculoperitoneal shunting. A pressure sustaining (PS) medical neonatal low-pressure valve (Medtronic Inc., Minneapolis, MN, USA) was chosen, appropriate for her age. Because of her genetic history, she also suffered from psychomotor impairment, epilepsy, scoliosis, and spastic quadriplegia.

Fifteen years after placement of the shunt, the patient was admitted to the emergency department of our hospital with increased frequency of epileptic seizures over the previous 2 months, as well as progressive drowsiness, nausea, and vomiting. Up to this point, she had never had a shunt revision. At physical examination, there were no new focal neurological deficits other than her known quadriplegia and mental retardation. A swelling was noticed behind the right ear, around the shunt valve.

**Fig. 2-3:**
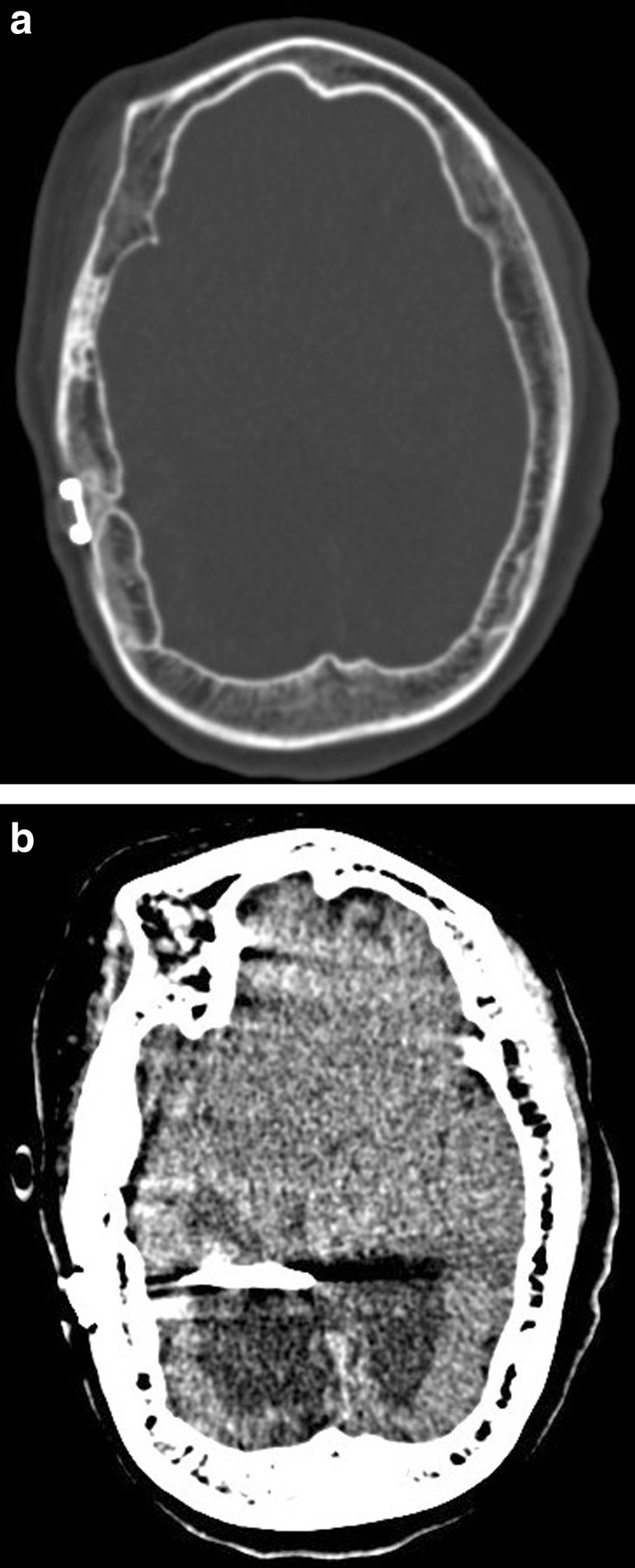
CT scan in bone and brain setting showing a bilateral calcified subdural mass with a thin hypodense area between the inner and outer rims

Suspecting a shunt dysfunction, we performed a CT and an X-ray shunt series. CT images showed an increased hydrocephalus and the shunt series showed a disconnection of the valve. More surprisingly, the CT also revealed a subdural ossified mass along both convexities. The brain seemed to be surrounded with another layer of bone and between the two layers there was just a narrow hypodense area (Figs. 1b and 2). In the bone setting, it almost looks like the skull is twice as thick as it should be (Fig. 3).

The patient underwent a surgical revision of the shunt, which was replaced with a complete new system, the Paedi GAV 9/29 (Miethke, Melsungen, Germany), more appropriate for her age at that time. After surgery, the clinical condition of the patient improved considerably. The patient was more reactive, no epileptic seizures were observed during the hospitalization, and she basically recovered to her level of functioning. The bilateral ossified masses were managed conservatively as they did not affect her neurological status.

## Discussion

Subdural hematomas and hygromas are a well-documented late complication of ventriculoperitoneal shunting [6]. Calcification and ossification of those are less common and the pathogenesis as well as treatment is still a matter of debate [7]. Bilateral subdural calcified masses may also be found after a trauma [8] or a meningitis [1, 9], but most of the cases are described in patients with a hydrocephalus shunting [10].

The most accredited theory correlates the formation of subdural hematomas or hygroma to overdrainage [11]. Interestingly, calcified subdural hematomas have never been reported as a complication of endoscopic third ventriculostomy [12]. This supports the correlation between overdrainage and formation of subdural hygroma and hematoma.

The calcification, followed by complete ossification, would be the result of a long-existing hematoma or hygroma [12]. Incomplete or insufficient absorption, possibly due to poor circulation or metabolic anomalies, would lead to stagnation of blood and ultimately to calcification [13, 14].

Surgical excision might be necessary when the calcification of the subdural mass causes a rise of intracranial pressure [5]. Still, the risk of an acute subdural hematoma or damage to the underlying brain tissue should always be taken into account before choosing surgical treatment [15]. In the presented case, the patient’s symptoms were correlated with the shunt dysfunction, not to the subdural calcified masses. However, the radiological finding of a matrioska head indicated a long-standing negative pressure gradient in the ventricle of our patient. Therefore, we not only decided to replace the shunt with a functioning one but also with a different valve. In cases where the etiology of the armored brains is overdrainage, a shunt revision should be considered [1]. To date, no study has shown the effect of a shunt revision on the subdural masses, but certainly it offers a simpler procedure with less morbidity than a craniotomy with drilling of the membranes. After surgical revision of the shunt, the patient recovered to her premorbid level of functioning, without the need of a risky intervention.

## Conclusions

Armored brain is a rare complication of long-existing subdural hematoma or hygroma due to overdrainage in patients treated for hydrocephalus. Even though it can possibly lead to mass-effect in some patients, our case demonstrated that such calcifications do not always affect the neurological status. Radiological findings of an armored brain in patients with a shunt should be considered as a sign of overdrainage, which should be appropriately treated.

The patient’s kin has consented to submission of this case report to the journal.
